# 

**Published:** 1862-11

**Authors:** 


### BOOKS RECEIVED.

*Transactions of the New York Academy of Medicine from June to July,* 1862, *containing valuable papers upon the following subjects, and the discussions thereon:*

Discussion of Dr. Conant's Paper on the science, causes and anatomical peculiarities of Human Monstrosities; Dr. J. Byrne on Pelvic Hæmatocele; Dr. M. H. Ranney upon Epilepsy; Dr. Noiggerath on Inversion of the Uterus; Dr. J. Marion Sims on Vaginismus; Dr. A. K. Gardner on Amputation of the Cervix Uteri; Dr. Alonzo Clark on Albuminuria.

*A Clinical Treatise on Diseases of the Liver; by* Dr. Fried. Theod. Frerichs, *Professor of Clinical Medicine in the University of Berlin, etc.; Medical Privy-Counsellor and Medical Adviser to the Ministry of Public Instruction and Medicine at Berlin, in two volumes, volume 2d. Translated by* Charles Murchison, M. D., F. R. C. P., *Physician to the London Fever Hospital, Lecturer on Pathological Anatomy, and Assistant Physician at the Middlesex Hospital. The New Sydenham Society, London,* 1861.

*A Year-Book of Medicine, Surgery, and their allied Sciences, for* 1861. *Edited by* Dr. Harley, Dr. Handfield Jones, Mr. Hulke, Dr. Graily Hewitt, *and* Dr. Sanderson, *for the New Sydenham Society. London,* 1862.

*Health: its Friends and its Foes; by* R. D. Mussey, M. D., LL. D., *late Professor of Anatomy and Surgery at Dartmouth College, N. H., and of Surgery in the Medical College of Ohio; Fellow of the American Academy of Arts and Sciences, etc., etc. Boston:* Gould & Lincoln, 1862.

*The Hospital Steward's Manual, for the instruction of Hospital Stewards, Ward-Masters, and Attendants, in their several duties; prepared in strict accordance with existing regulations and the customs of service in the Armies of the United States of America, and rendered authoritative by order of the Surgeon-General; by* Joseph Janvier Woodward, M. D., *Assistant Surgeon U. S. A., Member of the Academy of Natural Sciences of Philadelphia, etc. Philadelphia:* J. B. Lippincott & Co., 1862.

*Anatomy of the Arteries of the Human Body, Descriptive and Surgical, with the Descriptive Anatomy of the Heart; by* John Hatch Power, M. D., *Fellow, and Member of Council, of the Royal College of Surgeons; Professor of Descriptive and Practical Anatomy in the Royal College of Surgeons; Surgeon to the City of Dublin Hospital, etc. Authorized and adopted by the Surgeon-General of the United States Army for use in Field and General Hospitals. Philadelphia:* J. B. Lippincott & Co., 1862.

*The Action of Medicines in the System; or, "on the mode in which Therapeutic agents introduced into the Stomach produce their peculiar effects on the Animal Economy." Being the Prize Essay to which the Medical Society of London awarded the Fothergillian Medal for* 1862; *by* Frederick William Headland, M. D., B. A., F. L. S., *Licentiate of the Royal College of Physicians, etc., etc. Fourth American Edition. Philadelphia:* Lindsay & Blakiston, 1863.

For sale by Breed, Butler & Co. Price $2.

